# The Secretion of IL-22 from Mucosal NKp44^**+**^ NK Cells Is Associated with Microbial Translocation and Virus Infection in SIV/SHIV-Infected Chinese Macaques

**DOI:** 10.1155/2014/387950

**Published:** 2014-12-16

**Authors:** Wei Wang, Fangxin Wu, Zhe Cong, Kejian Liu, Chuan Qin, Qiang Wei

**Affiliations:** Key Laboratory of Human Diseases Comparative Medicine, Ministry of Health, Key Laboratory of Human Diseases Animal Models, State Administration of Traditional Chinese Medicine, Institute of Laboratory Animal Science, Chinese Academy of Medical Sciences and Comparative Medical Center, Peking Union Medical College, No. 5 Panjiayuan Nanli, Chaoyang District, Beijing 100021, China

## Abstract

Microbial translocation (MT) causes systemic immune activation in chronic human immunodeficiency virus (HIV) infection. The role of a novel subtype of innate lymphoid cells, the NKp44^+^ NK cells, in HIV/simian immunodeficiency virus- (SIV-) induced MT remains unknown. In this study, 12 simian-human immunodeficiency virus- (SHIV-) infected macaques were chosen and split into two groups based on the MT level. Blood and Peripheral lymphoid tissue were sampled for flow cytometric analysis, viral load detection, and interleukin testing. Then, six naive Chinese macaques were used to determine the dynamics of cytokine secretion from mucosal NKp44^+^ NK cells in different phases of SIV infection. As a result, the degranulation capacity and IL-22 production of mucosal NKp44^+^ NK cells were associated with the MT level in the SHIV-infected macaques. And the number of mucosal NKp44^+^ NK cells and IL-22 secretion by these cells were lower in the chronic phase than in the early acute phase of SIV infection. The number of mucosal NKp44^+^ NK cells and interleukin-22 (IL-22) secretion by these cells increased before MT occurred. Therefore, we conclude that a decline in IL-22 production from mucosal NKp44^+^ NK cells induced by virus infection may be one of the causes of microbial translocation in HIV/SIV infection.

## 1. Introduction

Chronic immune activation in gut-associated lymphoid tissue (GALT) caused by human immunodeficiency virus (HIV) infection has a severe impact on viral replication and disease progression. However, microbial translocation (MT), which is the leaking of commensal bacteria from the gut into systemic circulation, is a cause for systemic immune activation in chronic HIV infection [1]. MT from the gastrointestinal (GI) tract, which exceeds the capacity to clear the translocated microbial constituents, helps drive pathological immune activation, amplifies the inflammatory response, and alters the immune status [2]. Lipopolysaccharide (LPS), a major component of Gram-negative bacterial cell walls and a potent immunostimulatory product [3], can be quantitatively assessed in the plasma. LPS-binding protein (LBP) is produced by gastrointestinal and hepatic epithelial cells in response to LPS stimulation [1]. Plasma LPS and LBP levels are usually measured to determine the degree of MT in chronically HIV-infected individuals and in simian immunodeficiency virus- (SIV-) infected rhesus macaques [1, 2, 4]. Furthermore, MT in HIV-infected individuals may result from the loss of T helper 17 cells (T_H_17 cells) and decreased clearance of microbial products by phagocytosis, in particular damaged epithelial barrier [5]. Intestinal epithelial damage, caused by loss of intestinal epithelial cells (enterocytes) and disruption of tight junctions between the cells, may lead to increased microbial translocation in many diseases, including HIV infection [5]. Recent reports also indicate that a combination of structural epithelial deterioration and mucosal immunodeficiency is critical in driving HIV disease progression [2, 6], yet little is known about why the epithelial barrier breaks down and how this leads to MT.

Innate lymphoid cells (ILCs) represent a novel family of effector lymphocytes, which represent the first line of defense against virally infected cells and neoplastic cells [7, 8]; their loss in the gut may contribute to loss of intestinal mucosal integrity and disease progression in HIV/SIV infection [8]. As a major subset of ILCs, NK cells have an important role in eliminating HIV-1-infected target cells and controlling acquired immunodeficiency syndrome (AIDS) progression [9–11]. Several lines of evidence suggest that dramatic changes occur within the NK cell compartment during HIV infection, including phenotypic and functional changes [12–14]. SIV infection drives a shift in NK cell function that is characterized by decreased cytokine production, expanded cytotoxicity, and trafficking away from secondary lymphoid organs [15]. In addition, chronic immune activation may contribute to loss of functional potency of NK cells in HIV-1 infection, but elevated plasma LPS alone does not account for chronic activation and receptor loss in NK cells from HIV-1-infected individuals [16].

Interleukin- (IL-) 22 is a cytokine with epithelial reparative and regenerative properties that is produced by Th22 cells and other immune cell subsets [17]. At mucosal surfaces, IL-22 provides innate immune protection against bacterial and fungal infections, promotes inflammation, and enhances epithelial proliferation and repair [17, 18]. Even though IL-22 is produced mainly by CD4^+^ T cells, all mucosal IL-22-producing T cell subsets have been reported to be depleted very early during HIV or SIV infection [17, 19]. Recent studies have identified a novel subtype of ILCs, the NKp44^+^ NK cells, which have been generally designated as NK-22 cells based on their ability to secrete IL-22, IL-26, and leukemia inhibitory factor. This cell type is selectively localized in the tonsil and the gut mucosa and provides an innate source of IL-22 that may help constrain inflammation and protect mucosal sites [20]. However, the role of classic NK cells and NKp44^+^ NK cells in MT induced by HIV/SIV remains unknown. NKG2A, also known as NKG2 (CD159A), is a member of the killer cell lectin-like receptor family. This family is a group of transmembrane proteins that are preferentially expressed in NK cells.

In this study, we investigated the relationship between MT and NKp44^+^ NK cells in SIV/SHIV-infected Chinese macaques model. By comparing the number and functional potency of the NKp44^+^ NK cells of MT^high^ group and MT^low^ groups, including blood, peripheral lymph node, and lower ileum, we explored the role of NKp44^+^ NK cells in MT. Furthermore, in the second part of the study, we explored the dynamics of cytokine secretion by mucosal NKp44^+^ NK cells in the different phases of SIV infection. These findings suggest a role for NKp44^+^ NK cells in MT during chronic SIV/SHIV infection, which contributes to mucosal pathogenesis in HIV infection.

## 2. Results

### 2.1. MT Is Not Associated with Plasma Viral Load, CD4^+^ T Cell Count, Presence of Infectious Virus, or Route of Infection in the SHIV-Infected Chinese Macaques

LPS is an integral part of Gram-negative bacteria, whereas LBP is produced in response to LPS stimulation. LPS and LBP have been used as clinical markers of MT in several disease settings, such as inflammatory bowel disease [21], HIV-1, and SIV infection [1, 4]. In this study, the LPS levels of 20 SHIV-infected Chinese macaques varied from 0.085 to 0.850 EU/mL. The LBP levels of these 20 Chinese macaques varied from 1.952 to 9.554 mg/mL. The level of LBP was correlated with LPS (*R*_*s*_ = 0.527) (Table 1 and Figure  S1(a) in Supplementary Material available online at http://dx.doi.org/10.1155/2014/387950). To prevent any bias, the LPS and LBP levels of 20 SHIV-negative naïve Chinese macaques were also determined. The normal range of LPS in these animals was 0.09–0.19 EU/mL, and that of LBP was 0.968–3.884 mg/mL. Therefore, the LPS levels of 14 of the 20 SHIV-infected Chinese macaques were within this reference range, and the LBP levels of seven were within the reference range.

**Figure 1:**
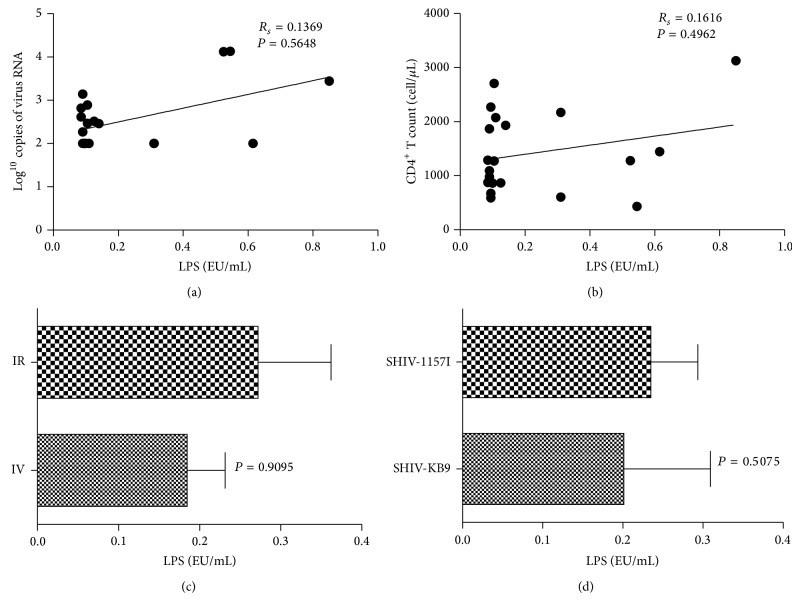
Lipopolysaccharide (LPS) levels are not associated with plasma viral load (PVL), CD4^+^ T cells, route of infection, or virus strain. Peripheral LPS levels of infected Chinese macaques did not correlate with (a) PVL (Spearman correlation test, *R*_*s*_ = 0.1369), (b) peripheral CD4^+^ T cells (Spearman correlation test, *R*_*s*_ = 0.1616), (c) infectious route (two-sided nonparametric Mann-Whitney *U* test, *P* = 0.9095), or (d) virus strain (two-sided nonparametric Mann-Whitney *U* test, *P* = 0.5075).

We determined plasma viral RNA loads and peripheral CD4^+^ T cell count to assess set point viremia and disease progression. The results showed that the majority of Chinese macaques were in the chronic phase of SHIV infection (Table 1). The levels of neither LPS nor LBP showed a strong correlation with plasma viral RNA loads and peripheral CD4 T cell counts (Figure 1 and Figure  S1). Furthermore, the levels of LPS and LBP were not correlated with presence of infectious virus and route of infection (Figure 1 and Figure  S1). In addition, the repeated dosing of 10 animals showed a possible association with MT, but the correlation rank-order coefficient was low (*R*_*s*_ = 0.2059). Based on the levels of LPS and LBP, six Chinese macaques with the lowest levels of MT (MT^low^) and six Chinese macaques with the highest level of MT (MT^high^) were chosen from the original group of 20 (Table 1 and Figure  S1(a)) for further research. The LPS and LBP levels of the MT^low^ group were not significantly different from those of the naïve Chinese macaques (*P* = 0.066 for LPS and *P* = 0.568 for LBP) but were significantly lower than those of the MT^high^ group (*P* = 0.004 for LPS and *P* = 0.003 for LBP) (Figures  S1(b) and  S1(c)).

### 2.2. Frequency of CD4^−^CD8^−^ T Cells in Peyer's Patches Is Associated with MT in the SHIV-Infected Chinese Macaques

**Figure 2:**
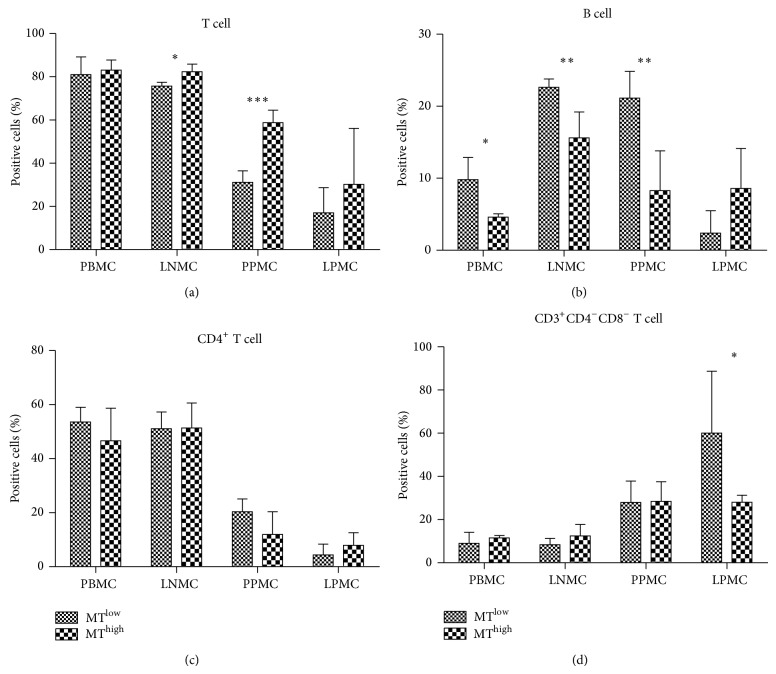
Comparison of the percentages of T cells and B cells at different sites in the microbial translocation (MT)^high^ and MT^low^ groups. (a) T cells, (b) B cells, (c) CD4^+^ T cells, and (d) CD3^+^CD4^−^CD8^−^ T cells of peripheral blood mononuclear cells (PBMCs), lymph node mononuclear cells (LNMCs), Peyer's patches mononuclear cells (PPMCs), and lamina propria mononuclear cells (LPMCs) were compared between the MT^high^ and MT^low^ groups of monkeys. The column bar indicates the mean of the ratio of target cells (lymphocytes in (a) and (b); T cells in (c) and (d)). Error bars and individual standard deviations (SD) are shown. ^*^ *P* ≤ 0.05, ^**^ *P* ≤ 0.01, and ^***^ *P* ≤ 0.001 as calculated by two-sided nonparametric Mann-Whitney *U* test.

As little is known about the effects of MT on lymphocyte subtypes in SHIV-infected Chinese macaques, we quantified the lymphocyte subtypes (including CD3^+^ T cells and CD20^+^ B cells) in the blood, ileum, and lymph node specimens. Representative results from the gating strategy used to identify lymphocytes in peripheral blood mononuclear cells (PBMCs) are shown in Figure  S2(a). The level of T cells in the MT^high^ group was higher than that in the MT^low^ group for both the lymph node (*P* < 0.05) and Peyer's patches (*P* < 0.001) specimens (Figure 2(a)), and the frequency of B cells in the MT^high^ group was lower than that in the MT^low^ group for the blood (*P* < 0.05), lymph node (*P* < 0.01), and Peyer's patches (*P* < 0.01) specimens (Figure 2(b)). These results indicated that MT can affect lymphocyte subtype in lymph node and Peyer's patches of Chinese macaques. However, further analysis showed that there was no significant difference in the ratio of CD3^+^CD4^+^ T cells and CD3^+^CD8^+^ T cells detected from the MT^low^ and MT^high^ groups (Figure 2(c) and data of CD8^+^ cells not shown). The frequency of CD3^+^CD4^−^CD8^−^ T cells in the MT^high^ group was higher than that in the MT^low^ group for the Peyer's patches (Figure 2(d)).

### 2.3. Number and Cytolytic Functions of CD56^+^CD16^−^ Cell Subtype in Peyer's Patches Mononuclear Cells (PPMCs) Are Enhanced in the MT^high^ Group in the SHIV-Infected Chinese Macaques

**Figure 3:**
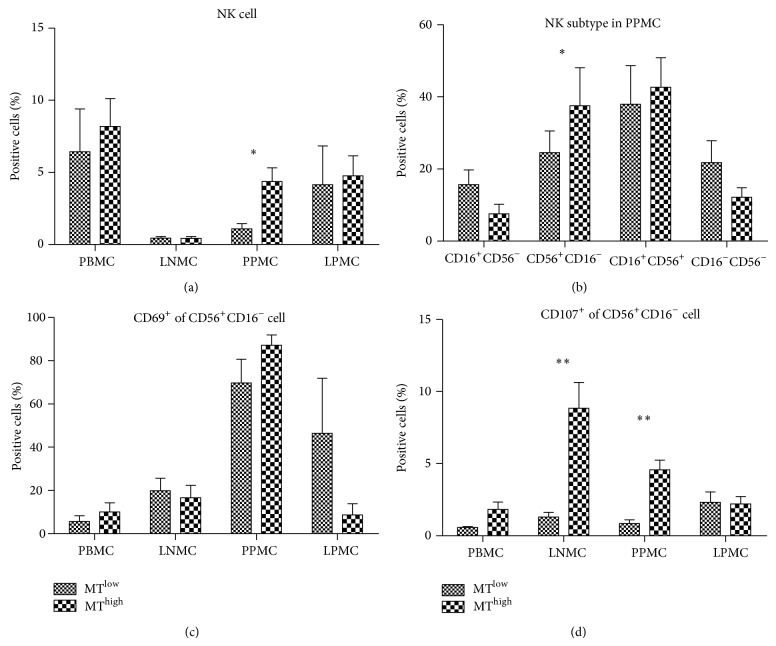
Numerical, phenotypical, and functional analyses of natural killer (NK) cells and the subtypes of different tissues from the microbial translocation (MT)^high^ and MT^low^ groups. (a) Distribution of macaque NK cell subsets in blood and tissues from the MT^high^ and MT^low^ groups. (b) NK cell subtype in Peyer's patches mononuclear cells (PPMCs) of the MT^high^ and MT^low^ groups. (c) Expression of CD69 and (d) CD107a molecules on CD56^+^ NK cell subsets of the MT^high^ and MT^low^ groups. The column bar indicates the mean of the ratio of target cells (mononuclear cells in (a); NK cells in (b); CD56^+^CD16^−^ NK cells in (c) and (d)). Error bars and individual standard deviations (SD) are shown. ^*^ *P* ≤ 0.05 and ^**^ *P* ≤ 0.01 as calculated by two-sided nonparametric Mann-Whitney *U* test.

Many lines of evidence have suggested that NK cells contribute to control of HIV/SIV infection [15, 22]. To assess the relationship between MT and NK cells, we determined the frequency of NK cells and the subtypes of PBMCs, lymph node mononuclear cells (LNMCs), PPMCs, and lamina propria mononuclear cells (LPMCs) from the MT^high^ (*n* = 6) and MT^low^ (*n* = 6) groups of Chinese macaques. Representative results from the gating strategy used to identify NK cell subtypes in PBMCs are shown in Figure  S2(b). The distribution of NK cells was perturbed in different tissues of SHIV-infected Chinese macaques, with the fraction being higher in PBMCs and lower in LNMCs (Figure 3(a)). The frequency of NK cells in PPMCs was higher in the MT^high^ group than in the MT^low^ group (*P* < 0.05) (Figure 3(a)). Analysis of the mucosal NK cell subpopulations using the CD16 and CD56 markers showed that in PPMCs the frequency of CD56^+^CD16^−^ cells was higher in the MT^high^ group (*P* < 0.05) (Figure 3(b)). Analysis of the function of NK subtypes showed that CD69 expression of CD56^+^CD16^−^ cells of PPMCs in MT^high^ and MT^low^ groups was similar (Figure 3(c)), but that the CD107 expression of CD56^+^CD16^−^ cells of PPMCs in the MT^high^ group was remarkably higher than in the MT^low^ group (*P* < 0.01) (Figure 3(d)), suggesting that the cells in the MT^high^ group had increased cytolytic function. Furthermore, the CD107 expression of CD56^+^CD16^−^ cells in the LNMCs was remarkably higher in the MT^high^ group than in the MT^low^ group (*P* < 0.01) (Figure 3(d)), suggesting that NK cells at the immune effector site had increased cytolytic function in the presence of MT.

### 2.4. Degranulation Capacity and IL-22 Production of Mucosal NKp44^+^ NK Cells Are Associated with the Level of MT in the SHIV-Infected Chinese Macaques

**Figure 4:**
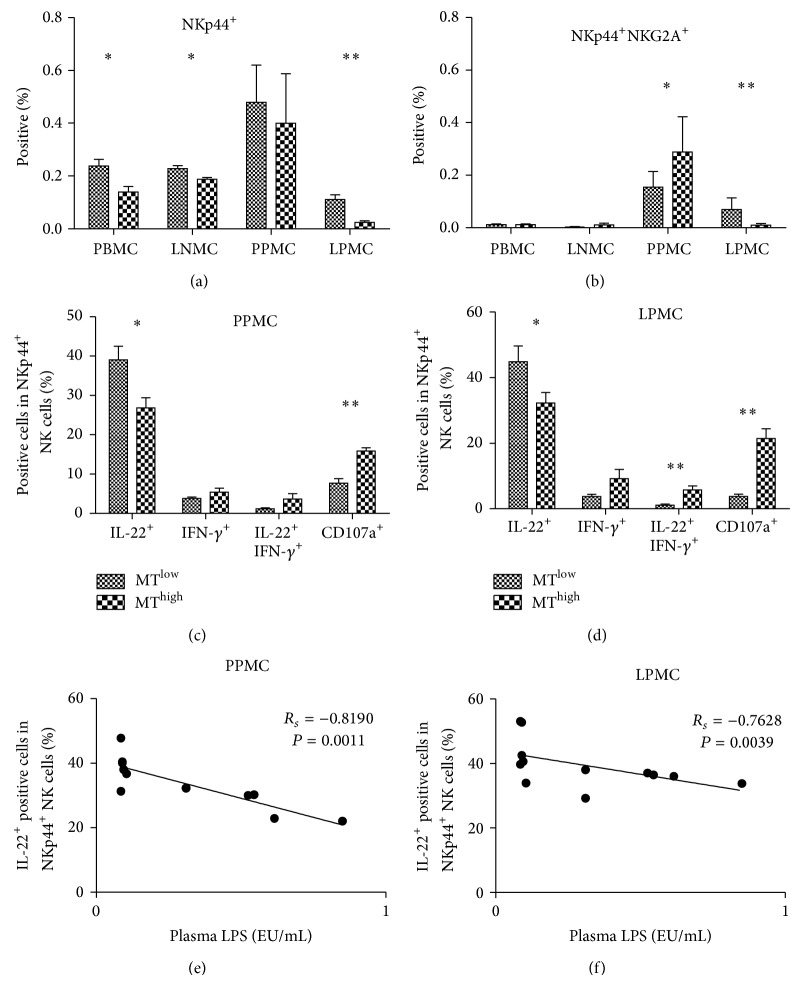
Percentage and function of natural killer (NK)p44^+^ cell subsets are altered in the microbial translocation (MT)^high^ and MT^low^ groups. Frequency of (a) NKp44^+^ NK cells and (b) NKG2A^+^NKp44^+^ NK cells in peripheral and mucosal tissues from the MT^low^ and MT^high^ groups. The expressions of IL-22, IFN-*γ*, and CD107a of NKp44^+^ NK cells were compared between the MT^low^ and MT^high^ groups in (c) Peyer's patches mononuclear cells (PPMCs) and (d) lamina propria mononuclear cells (LPMCs). The column bar indicates the mean of the ratio of target cells (mononuclear cells in (a) and (b); NKp44^+^ NK cells in (c) and (d)). Error bars and individual standard deviations (SD) are shown. Spearman correlation test showed that the number of IL-22^+^NKp44^+^ NK cells in PPMCs and LMPCs correlated well with plasma lipopolysaccharide (LPS) levels. ^*^ *P* ≤ 0.05 and ^**^ *P* ≤ 0.01 as calculated by two-sided nonparametric Mann-Whitney *U* test.

Representative results of the gating strategy used to identify NKp44^+^ NK cells in PBMCs are shown in Figure  S2(c). A modest reduction in the frequency of NKp44^+^ NK cells was observed for the MT^high^ group compared with the MT^low^ group in terms of PBMCs (*P* < 0.05), LNMCs (*P* < 0.05), and LPMCs (*P* < 0.01) (Figure 4(a)); however, the standard deviation (SD) values of NKp44^+^ NK cells in PPMCs were high in the MT^high^ and MT^low^ groups. Interestingly, we found NKp44^+^NKG2A^+^ double-positive cells in all of the Chinese macaques examined in this study. This novel cell subtype was appropriately localized mainly to the mucosa of the intestinal tract. The frequency of this subtype in PPMCs of the MT^high^ group was higher than that in the MT^low^ group (*P* < 0.05) but followed the reverse trend in LPMCs (*P* < 0.01) (Figure 4(b)).

In the next analysis, functional alterations in NKp44^+^ NK cells were examined. IL-22 levels of NKp44^+^ NK cells in PPMCs and LPMCs from the MT^high^ group were lower than those from the MT^low^ group (*P* < 0.05). Conversely, CD107a levels of NKp44^+^ NK cells in PPMCs and LMPCs from the MT^high^ group were higher than those from the MT^low^ group (*P* < 0.01). In the LPMCs, the frequency of IL-22^+^IFN-*γ* ^+^ double-positive NKp44^+^ NK cells was higher in the MT^high^ group (*P* < 0.01). Spearman correlation test showed that the number of IL-22^+^ NKp44^+^ NK cells in PPMCs (*R*_*s*_ = −0.8190) and LMPCs (*R*_*s*_ = −0.7628) correlated well with plasma LPS and LBP levels (Figures 4(e) and 4(f)). Thus, NKp44^+^ NK cells were functionally altered, expressing CD107a instead of IL-22 in PPMCs and in LPMCs. Moreover, MT induced increased CD107a and IL-22^+^IFN-*γ* ^+^ phenotypes of NKp44^+^ NK cells.

### 2.5. MT Does Not Induce IL-10 and IL-22 in the Gut in the SHIV-Infected Chinese Macaques

NKp44^+^ NK cells provide an innate source of IL-22 that may help constrain inflammation and protect mucosal sites [20]. Therefore, IL-22 levels were determined in the plasma and the gut tissue. IL-22 plasma levels were beneath the threshold of detection for both groups of Chinese macaques, and the IL-22 levels detected in the gut were not statistically different between the MT^high^ and MT^low^ groups (data not shown). The cytokines secreted by NK-22 cells are capable of stimulating epithelial cells to secrete IL-10, to proliferate, and to express a variety of mitogenic and antiapoptotic molecules [20]. Similar to the IL-22 results, IL-10 was not detected in the plasma but was detected in the gut tissue, at levels that were similar between the MT^high^ group and MT^low^ group. Thus, the differential functional alteration of NKp44^+^ NK cells that occurred in the MT^high^ and MT^low^ groups did not affect the level of effector cytokines expressed by these cells.

### 2.6. Number of Mucosal NKp44^+^ NK Cells and Their Cytolytic Functions Decreased during the Course of the Infection in the SIVmac239-Infected Macaques

**Figure 5:**
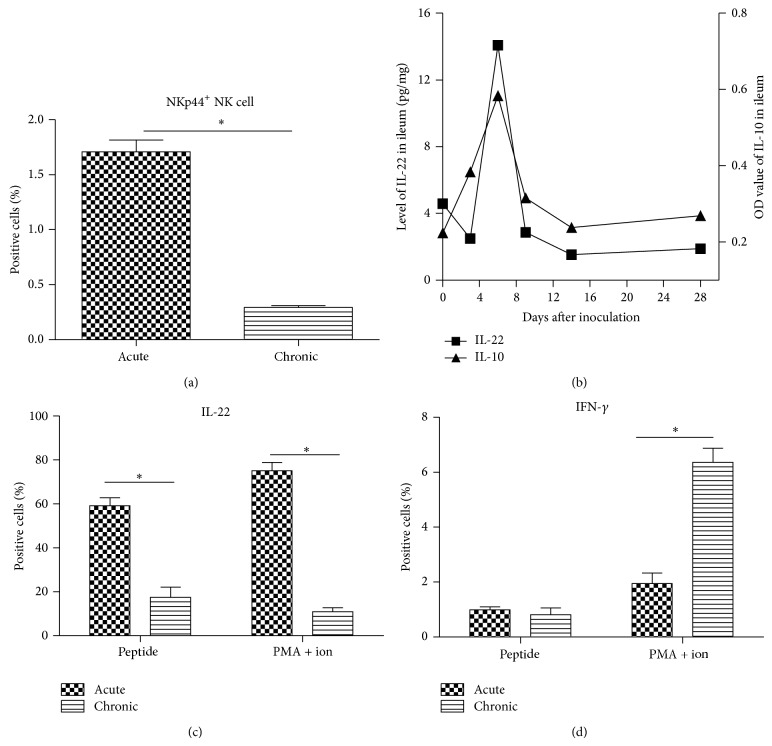
Number and cytolytic function of mucosal natural killer (NK)p44^+^ NK cells during early acute and chronic phases of simian immunodeficiency virus (SIV) infection. (a) Frequency of NKp44^+^ NK cells at early acute and chronic phases of SIV infection. (b) Levels of IL-22 and IL-10 in ileum of monkeys infected with SIV during the early acute phase. (c) IL-22 secretion and (d) IFN-*γ* secretion of mucosal NKp44^+^ NK cells stimulated with peptide or phorbol 12-myristate 13-acetate (PMA) + ionomycin. The column bar indicates the mean of the ratio of target cells (mononuclear cells in (a); NKp44^+^ NK cells in (c) and (d)). Error bars and individual standard deviations (SD) are shown. ^*^ *P* ≤ 0.05 as calculated by two-sided nonparametric Mann-Whitney *U* test.

To investigate the dynamics of cytokine secretion from mucosal NKp44^+^ NK cells in different phases of virus infection, the number and cytolytic functions of mucosal NKp44^+^ NK cells were compared between early acute and chronic phases of SIV infection in the second part of this study. Six naïve Chinese macaques were infected with SIVmac239 and the status of infection, plasma viral load, and CD4^+^ T cell number confirmed that a classic SIV infection had been established (Figure  S3). The mucosal NKp44^+^ NK cell count was higher in the early acute infection phase than in the chronic infection phase (*P* < 0.05) (Figure 5(a)), and the IL-22 secretion from mucosal NKp44^+^ NK cells was higher in the early acute phase than in the chronic phase (*P* < 0.05). Dual stimulation with phorbol 12-myristate 13-acetate (PMA) and ionomycin led to mucosal NKp44^+^ NK cells secreting more IL-22 in the early acute phase than in the chronic phase. The mucosal NKp44^+^ NK cells secreted mainly IL-22 in the early acute phase and IFN-*γ* in the chronic phase (Figures 5(c) and 5(d)). IL-22 and IL-10 levels in gut peaked at day 6 after inoculation and then decreased to normal levels (Figure 5(b)). In addition, IL-22 and IL-10 levels were lower than the detection limit in LNMCs and PBMCs from SIV-infected Chinese macaques.

### 2.7. SIV Infection Stimulates IL-22 Secretion of Mucosal NKp44^+^ NK Cells during Early Acute Phase Infection

**Figure 6:**
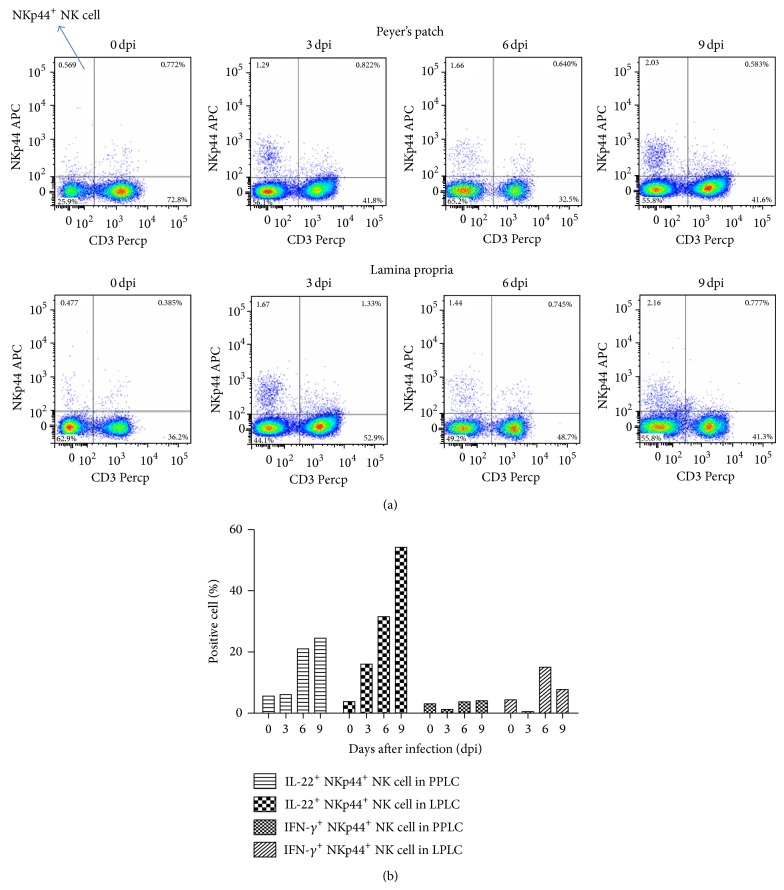
Number of and IL-22 secretion from mucosal natural killer (NK)p44^+^ NK cells increased in the early acute phase before occurrence of microbial translocation (MT). (a) Number of mucosal NKp44^+^ NK cells (upper left quadrant) at the early acute phase of simian immunodeficiency virus (SIV) infection. (b) IL-22 or IFN-*γ* secretion of mucosal NKp44^+^ NK cells during early acute phase of SIV infection.

Reeves et al. have demonstrated that SIV infection was accompanied by depletion of NKp44^+^ NK cells as well as an altered functional profile of the remaining cells characterized by decreased IL-17 secretion, increased IFN-*γ* secretion, and, surprisingly, increased cytotoxic potential [23]; however, the authors did not address the effect of MT. Therefore, to determine whether virus infection or MT led to depletion of mucosal NKp44^+^ NK cells and modification of the functional repertoire of the subtype, the kinetics of mucosal NKp44^+^ NK cells were determined during the early acute phase of virus infection and before the occurrence of MT in the second part of the study. The ratio of CD3^−^ NKp44^+^ NK cells in the lymphocytes increased gradually from 0.57% (0 dpi) to 2.03% (9 dpi) in the Peyer's patches and from 0.48% (0 dpi) to 2.16% (9 dpi) in the lamina propria (Figure 6(a)). Cytokine secretion was also analyzed from NKp44^+^ NK cells, and IFN-*γ* secretion was found to decline from 3.09% (0 dpi) to 1.23% (3 dpi) and then increase to 4.10% (9 dpi) in the Peyer's patches. In the lamina propria, IFN-*γ* secretion declined from 4.38% (0 dpi) to 0.53% (3 dpi) and then increased to 15.00% (6 dpi) and 7.74% (9 dpi). It is noteworthy that IL-22 secretion in the mucosal NKp44^+^ NK cells increased dramatically with virus infection, from 5.58% (0 dpi) to 24.50% (9 dpi) in the Peyer's patches and from 3.78% (0 dpi) to 54.20% (9 dpi) in the lamina propria (Figure 6(b)). These data clearly show that SIV infection causes an increased secretion of IL-22 from mucosal NKp44^+^ NK cells during the early acute phase.

## 3. Discussion

The 20 SHIV-infected Chinese macaques evaluated in the first part of this study represented the plateau stage of the infectious phase. Although SHIV infection did not cause CD4^+^ T cell loss in peripheral blood, a lower level of CD4^+^ T cells was found in the lymph node and the gut tissue of SHIV-infected Chinese macaques when compared to naïve Chinese macaques (an average of 68% CD4^+^ T cells in the lymph node and 31% in the gut tissue, unpublished data). The SHIV infection also led to a slow but persistent decline in CD4^+^ T cell count. Therefore, these SHIV-infected Chinese macaques were characterized as slow progressors or controllers.

LPS is a heterogeneous collection of molecules that is found in the cell wall of most Gram-negative bacteria [24], and circulating LPS has been detected in hosts with Gram-negative bacterial infections. In particular, Brenchley et al. found that individuals with HIV infection and CD4^+^ T lymphocyte depletion had higher levels of plasma LPS than controls [1]. This finding has been confirmed by some studies on HIV and SIV infection [1, 25–29], but other studies have shown that LPS detection can be masked in undiluted serum and plasma samples. Therefore, MT may be substantially underestimated in some studies [30]. In the current study, we used the TAL assay to measure LPS levels in diluted plasma of infected Chinese macaques to accurately determine the extent of MT. As LPS levels increase during advanced viral infection even though they are highly variable, we were able to confirm the extent of MT by determining the plasma LBP concentration. Furthermore, we discovered a link between MT levels and infection parameters in experimentally infected Chinese macaques, including plasma viral load, CD4^+^ T cell count, presence of infectious virus, and route of infection. As reported previously [31], MT is independent of SIV plasma viral load. MT is considered one of the important factors capable of accelerating disease progression, but peripheral CD4^+^ T cells are not correlated with MT in the chronic phase of SHIV infection. Further analysis in our study indicated no links between the virus strains or route of infection and the level of MT.

HIV-1 can directly impair the function and integrity of the epithelial barrier, thereby allowing MT from the gut [6]. Moreover, the structural damage to the mucosal barrier, which causes MT, occurs prior to the depletion of memory CD4 T cell subsets in the ileum of Chinese macaques at an early stage of SIV infection (unpublished data). Therefore, we determined what T cell and B cell changes are caused by MT. CD3^+^ T cell ratio in peripheral lymph nodes and the mucosa Paasche lymph nodes of the MT^high^ group was significantly higher than that of the MT^low^ group, but CD4^+^ T cell and CD8^+^ T cell ratio of the MT^high^ group did not markedly increase following stimulation by microbial products. This data demonstrates that microbial products can induce the increase of CD4^−^CD8^−^ T cells in peripheral lymph nodes and Peyer's patches, but it remains unknown whether these increased double-negative T cells retain their functions as CD4^+^ T cells. It has been demonstrated that B cell apoptosis occurs in SIV-infected Chinese macaques with MT, suggesting that B cell death may be induced by HIV infection and MT [32]. In this study, we showed that B cell ratio of the MT^high^ group was significantly lower than that of the MT^low^ group in blood, peripheral lymph nodes, and the mucosal Peyer's patches, suggesting that B cell death may be induced by MT.

NK cells, which belong to the group of ILCs, are classically viewed as effector cells that kill virus-infected and neoplastic cells [23]. In this study, the cell count of CD56^+^CD16^−^ subtype was increased in Peyer's patches and enhanced the cytolytic function in Peyer's patches and lymph glands. Fehniger et al. first described NK cells in human lymph nodes as primarily CD56^+^CD16^−^ cells that could regulate development of adaptive immune responses [33]. CD56^+^ NK cells secrete copious amounts of cytokines with low levels of degranulation [15]. During chronic SIV infection, the number of CD56^+^ NK cells in peripheral blood was not significantly reduced [15]. We demonstrated that these cells had redistributed to tissues, such as the gut mucosa, upon stimulation by microbial products. Although SIV infection increased CD107a expression in stimulated CD56^+^ NK cells [34], CD107a^+^CD56^+^ NK cells were significantly increased in lymph nodes and Peyer's patches of the MT^high^ group. Thus, CD56^+^ NK cells at immune effector sites have increased cytolytic function as a result of MT.

During SIV infection, NKp44^+^ NK cells are depleted and have an altered functional profile [23]. In this study, we found that, as a result of MT, the number of NKp44^+^ NK cells decreased and IL-22 expression level in these cells also decreased, mainly in LPMCs. Reeves et al. reported the identification of two distinct lineages of mucosal NK cells, NKG2A^+^ and NKp44^+^ cells, and also found that NKG2A^+^ cells are more differentiated than NKp44^+^ NK cells [23]. Interestingly, we found a new cell subset of NKp44^+^ NK cells that express an inhibitory NK receptor, NKG2A. Therefore, this cell subtype may be an intermediate cell between NKG2A^+^ and NKp44^+^ cells.

Many different types of lymphocytes secrete IL-22 [35]. At mucosal sites, IL-22 is produced mainly by CD4^+^ T cells [19] and by a subset of mucosal NK cells that express the receptor NKp44 (NKp44^+^ NK cells) [36]. In this study, the number of NKp44^+^ NK cells and IL-22 expression level of these cells decreased as a result of stimulation by microbial products. Therefore, we conclude that CD4^+^ T cells or other cells secreted more IL-22 following MT, as has been previously reported [19]. The results for IL-10 secretion also led to a similar conclusion. Our primate study demonstrated that innate lymphoid cells, including NK cells and NKp44^+^ NK cells, play an important role in MT that results from SHIV infection.

As described in the Results section [23], it was not known whether virus infection or MT depletes and modifies the functional repertoire of mucosal NKp44^+^ NK cells. We chose SIV-infected Chinese macaques experiencing early acute infection without MT to study if a single virus infection can affect NKp44^+^ NK cells. With the exception of one animal at 8 dpi, Estes et al. found that only very low levels of LPS^+^ cells are within the lamina propria of rhesus macaques between 1 and 10 dpi. These observations are indistinguishable from those made with SIV-uninfected animals [2]. Therefore, we chose four time points (0, 3, 6, and 9 dpi) for analyzing cell count and function. LPS analysis demonstrated that MT did not occur in these six macaques during the observation period (data not shown). Thus, virus infection but not MT appears to be one of the causes of cell loss and IL-22 production by mucosal NKp44^+^ NK cells. Moreover, the mucosal NKp44^+^ NK cells progressively declined as the disease continued to develop.

In conclusion, a decline in IL-22 production from mucosal NKp44^+^ NK cells induced by virus infection may be one of the causes of MT in HIV/SIV infection. The collective findings of this study suggest a role for NKp44^+^ NK cells (a set of ILCs) in MT during chronic SIV/SHIV infection, whereby mucosal pathogenesis of HIV infection is facilitated.

## 4. Methods

### 4.1. Ethics Statement

The Institutional Animal Care and Use Committee (IACUC) of the Institute of Laboratory Animal Science, Chinese Academy of Medical Sciences (ILAS, CAMS), approved all procedures that were performed on macaques (Protocol Permit Number ILAS-VL-2011-005). ILAS facilities used in this study had been fully accredited by the Association for Assessment and Accreditation of Laboratory Animal Care International (AAALAS). This study was carried out in strict accordance with the recommendations of the IACUC Guide (established in 2006) and Weatherall report: “The use of non-human primates in research.”

### 4.2. Animals and Study Design (Infection and Sampling)

Twenty SHIV-infected Chinese macaques were used in the first part of this study and were obtained from a program called “establishment of SHIV-infected rhesus monkeys of Chinese origin model” (Table 1). For this study, Chinese macaques were infected intravenously or intrarectally with 5, 50, 500, or 1000 TCID_50_ SHIV-KB9 [37] or 5, 20, or 50 TCID_50_ SHIV-1157ipd3N4 virus [38, 39] (Table 1). These viruses were kindly supplied by the National Institutes of Health (NIH) AIDS Research and Reference Reagent Program (https://www.aidsreagent.org/). Blood samples were collected from all the animals and analyzed for plasma LPS and LBP level at 425 days after inoculation. Then, twelve Chinese macaques were euthanized for tissue sampling based on the plasma LPS and LBP levels. Blood samples and lymph nodes were collected, along with 8–10 cm of the lower ileum tissue by surgical resection.

Another six naïve Chinese macaques were used in the second part of the study and tested negative for SRV, STLV, SIV, monkey B virus, and TB. These six Chinese macaques were inoculated intravenously with 1 mL of viral suspension containing 10^4^ TCID_50_ of SIVmac239 (a generous gift from P.A. Marx in 1994). Tissue samples from the ileum were collected at 0, 3, 6, 9, 210, 217, and 224 dpi by enteroscopy.

### 4.3. Detection of LPS and LBP

LPS was detected in heat-inactivated plasma samples using the Chromogenic End-Point Tachypleus Amebocyte Lysate (TAL) Test by Xiamen (Fujian, China) and following the manufacturer's protocol. Briefly, samples were diluted tenfold with endotoxin-free water and heated at 70°C for 10 min to inactivate interfering plasma components. After incubation with the TAL reagent and the chromogen, the absorbance of duplicate samples was measured at 545 nm in a photometric plate reader. LBP concentration in the samples was measured by enzyme-linked immunosorbent assay (ELISA) (HyCult Biotechnology, Uden, The Netherlands) according to the manufacturer's instructions. Briefly, 100 *μ*L of the diluted sample was added to its respectively coated ELISA plate. The plate was then washed four times with PBS, after which the biotinylated detection antibody (tracer and streptavidin-peroxidase and tetramethyl benzidine (TMB)) was added. Each sample was tested in duplicate and measured at 450 nm in a photometric plate reader.

### 4.4. Analysis of Viral RNA

Virus load was measured as previously described [40]. Briefly, viral RNA was isolated from plasma and ileum tissue, reverse transcribed, and amplified using a TaqMan real-time PCR technique (Gag91F: 5′-GCA GAG GAG GAA ATT ACC CAG TAC-3′ and Gag91R: 5′-CAA TTT TAC CCA GGC ATT TAA TGT T-3′) and an ABI 7500 sequence detection system (Applied Biosystems Inc., Foster City, CA, USA). Serial dilutions of* in vitro* transcripts of SIV* gag *were used to generate a standard curve for each run. The copy numbers were determined by automated interpolation onto the standard curve generated by the ABI 7500 software v2.0.5 (Applied Biosystems Inc.).

### 4.5. Mononuclear Cell Isolation

PBMCs and LNMCs were isolated using standard procedures. The method used for the identification of Peyer's patches and lamina propria was carried out as described previously [41]. Briefly, to detect gut mucosal Peyer's patches, the resected ileum was washed in phosphate-buffered saline (PBS), the tissue area was measured, the muscularis propria was removed with a scalpel, and the mucosal surface was stained with 1% methylene blue. Peyer's patches were cut free from the adjacent tissue, which was used as follicle-free control lamina propria for isolation of LPMCs. The dissected Peyer's patches were suspended in 0.9% NaCl and used to isolate the PPMCs.

The various cell types were isolated from intestinal tissues by using a combination of mechanical and enzymatic dissociation procedures, as previously described [42]. Briefly, PPMCs were isolated from intestinal segments by using EDTA and mechanical agitation, while LPMCs were isolated from the remaining intestinal pieces by collagenase digestion. Lymphocytes were enriched by Percoll density gradient centrifugation [43]. Intestinal cell viability was always >90%, as determined by trypan blue dye exclusion assay. In all cases, the cells were stained on the day of sampling, and cell suspensions were kept on ice between incubations to prevent any changes in expression of cell surface markers after the tissues had been harvested. Previous studies have shown that these procedures do not affect the expression of cell surface markers, including those associated with cell activation [42, 44]. A total of 5 × 10^6^ cells were used for flow cytometry assays.

### 4.6. Polychromatic Flow Cytometry

Flow cytometry analysis of the mononuclear cell surface and intracellular molecules was carried by standard protocols. Briefly, cells (10^6^ cells from each of the above-described samples) were incubated with appropriate amounts of monoclonal antibodies (see below) at 4°C for 30 min, followed by washing (400 ×g, 7 min) and fixation in 2% paraformaldehyde. Anti-CD3/PE-Cy7 (Catalogue Number SP34-2), anti-CD4/Percp-Cy5.5 (L200), anti-CD8/APC-Cy7 (RPA-T8), and anti-CD69/FITC (FN50) were purchased from BD Biosciences (La Jolla, CA, USA). Anti-CD16/Percp-Cy5.5 (3G8), anti-CD56/APC (MEM-188), anti-CD28/PE (CD28.2), anti-IFN-*γ*/APC-Cy 7 (4S.B3), and anti-CD56/APC (DX2) were purchased from BioLegend (San Diego, CA, USA). Anti-CD107a/PE (eBioH4A3) and anti-IL-22 (IL22JOP) were purchased from eBioscience (San Diego, CA, USA). The T cells, B cells, and NK cells were collectively gated by CD3, CD4, CD8, and CD20 markers. The NK cells and their classical subtypes were uniquely gated by CD3, CD8, NKG2A, CD16, and CD56 markers. The NKp44^+^ NK cell subtype was uniquely gated by CD3, CD8, and NKp44 markers. Before intracellular cytokine staining, the cells were stimulated for 16 h in the presence of Brefeldin A, SIV peptide pool (for antigen-specific stimulation), or PMA + ionomycin (for nonspecific stimulation). Isotype-matched controls and/or fluorescence-minus-one (FMO) controls were included in all assays. Acquisitions (≥50,000 lymphocytes) were made on a FACSCanto I flow cytometer (BD Biosciences, San Jose, CA, USA) and analyzed using FlowJo software 7.2 (Tree Star Inc., Ashland, OR, USA).

### 4.7. Cytokine Production Analysis

IL-17 (U-CyTech, Utrecht, The Netherlands) and IL-22 (BioLegend, San Diego, CA, USA) were measured using commercial ELISA kits. Briefly, 100 mg of ileum tissue, PBMCs, and lymph node tissues were homogenized with a tissue grinder. Then, 100 *μ*L of supernatant was added to the respective coated ELISA plate. The plate was washed three times with PBS and the biotinylated detection antibody (avidin-HRP and Substrate Solution F or tetramethyl benzidine (TMB)) was added. The samples were set up in duplicate and were measured at 450 nm in a photometric plate reader.

### 4.8. Statistical Analyses

All statistical and graphical analyses were performed using SPSS software version 11.5 (SPSS Inc., Chicago, IL, USA) and GraphPad Prism software (GraphPad Software, La Jolla, CA, USA). Nonparametric Wilcoxon matched pairs, Mann-Whitney, and Spearman correlation tests were used where appropriate, and *P* < 0.05 was considered statistically significant [45].

## Supplementary Material

The Supplementary material provides respectively Correlation of peripheral LPS levels and plasma LBP with MT (Fig. S1); Schematic representation of the gating strategy used to identify (Fig. S2); Status of infection, plasma viral loads, and CD4^+^ T cell numbers in SIVmac239-infected monkeys during study phase II (Fig. S3).

## Figures and Tables

**Table 1:** Summary of viruses and associated experimental parameters used in the first part of the study.

Animal number	Sex	Virus stock	Dose (TCID_50_)	Route of infection	Plasma viral load (copies/mL)	Peripheral CD4 T cells (cells/*μ*L)	Plasma LPS (EU/mL)	Plasma LBP (mg/mL)	Group
G1005V	♀	SHIV- 1157ipd3N4	50	IV	<100	606	0.310	9.554	MT^high^
G1006V	♀		50	IV	291	1271	0.105	2.667	MT^low^
G1007V	♀		50	IV	<100	2271	0.095	4.327	/
G1008V	♀		5	IV	<100	2172	0.310	9.554	MT^high^
G1009V	♀		5	IV	327	867	0.125	6.649	/
G1010V	♀		5	IV	<100	592	0.095	1.952	MT^low^

G1011V	♀	SHIV-KB9	500	IV	13240	1277	0.525	7.309	MT^high^
G1012V	♀		50	IV	<100	674	0.095	4.879	/
G1013V	♀		5	IV	<100	866	0.100	5.515	/
G1014V	♀		1000	IV	411	1287	0.085	2.562	MT^low^

G1015R	♀	SHIV- 1157ipd3N4	20	IR (repeated 4)	289	1931	0.140	4.718	/
G1016R	♂		20	IR (repeated 4)	660	875	0.085	2.590	MT^low^
G1017R	♂		20	IR (repeated 6)	1387	978	0.090	2.177	MT^low^
G1018R	♂		20	IR (repeated 5)	772	2707	0.105	5.988	/
G1019R	♀		20	IR (repeated 4)	13555	431	0.545	9.554	MT^high^
G1020R	♀		20	IR (repeated 10)	<100	2075	0.110	2.351	/
G1021R	♀		20	IR (repeated 10)	<100	1093	0.090	3.317	MT^low^
G1022R	♀		20	IR (repeated 4)	185	1869	0.090	6.195	/
G1023R	♂		20	IR (repeated 13)	<100	1446	0.615	7.058	MT^high^
G1024R	♂		20	IR (repeated 10)	2776	3127	0.850	9.554	MT^high^

IR: intrarectal; IV: intravenous; LBP: lipopolysaccharide-binding protein; LPS: lipopolysaccharide, MT: microbial translocation; SHIV: simian human immunodeficiency virus.
