# Supplementary material for: The Secretion of IL-22 from Mucosal NKp44+ NK Cells Is Associated with Microbial Translocation and Virus Infection in SIV/SHIV-Infected Chinese Macaques
Source: J Immunol Res. 2014 Dec 16;2014:387950. doi: 10.1155/2014/387950 (PMC4352435; doi:10.1155/2014/387950)
Supplement: Supplementary file 1 — The Supplementary material provides respectively Correlation of peripheral LPS levels and plasma LBP with MT (Fig. S1); Schematic representation of the gating strategy used to identify (Fig. S2); Status of infection, plasma viral loads, and CD4+ T cell numbers in SIVmac239-infected monkeys during study phase II (Fig. S3). [file 387950.f1.pdf]

Fig. S1. Correlation of peripheral LPS levels and plasma LBP with MT. (a) The levels of the LPS and LBP were correlated in infected monkeys (*n*=20). (b, c) Comparison of the level of (b) LPS and (c) LBP in naïve, MT^{low} and MT^{high} groups.

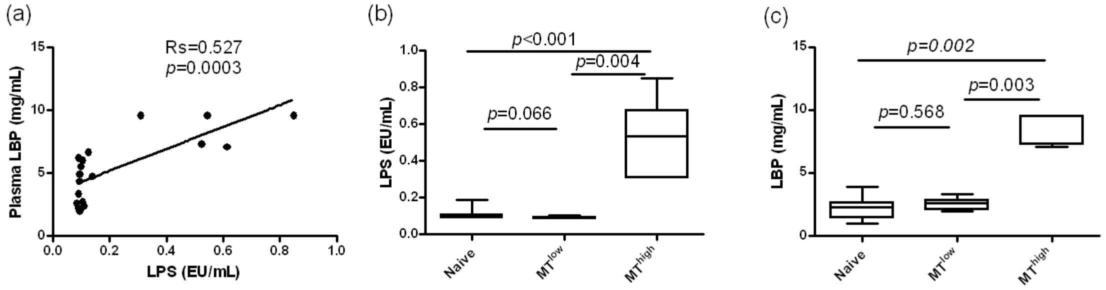

Fig. S2. Schematic representation of the gating strategy used to identify (a) lymphocytes, (b) NK cells, and (c) NKp44^{+} NK cells among mononuclear cells in PBMCs from experimental monkeys.

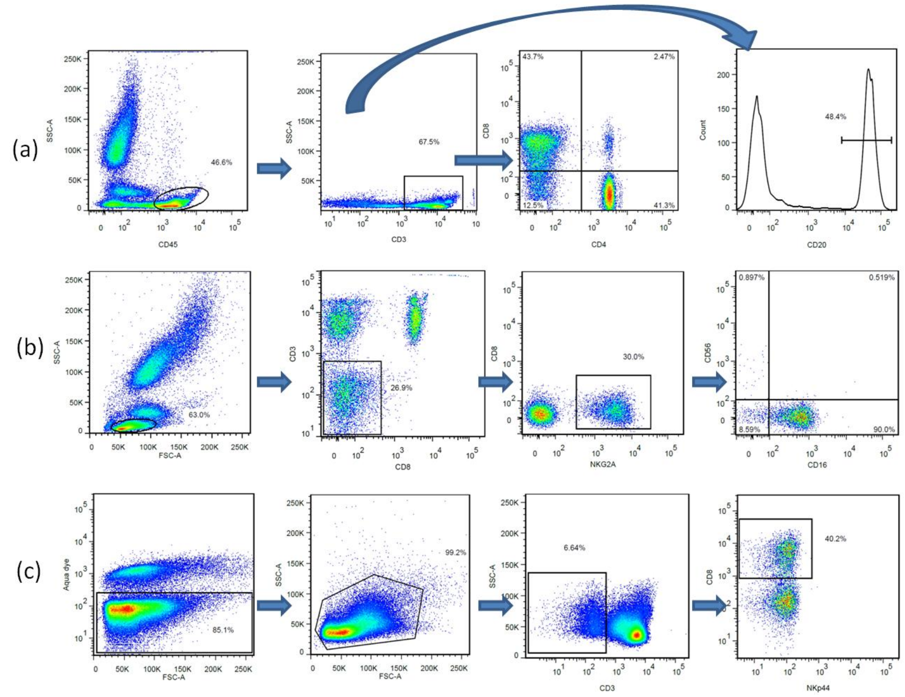

Fig. S3. Status of infection, plasma viral loads, and CD4^{+} T cell numbers in SIVmac239-infected monkeys during study phase II.

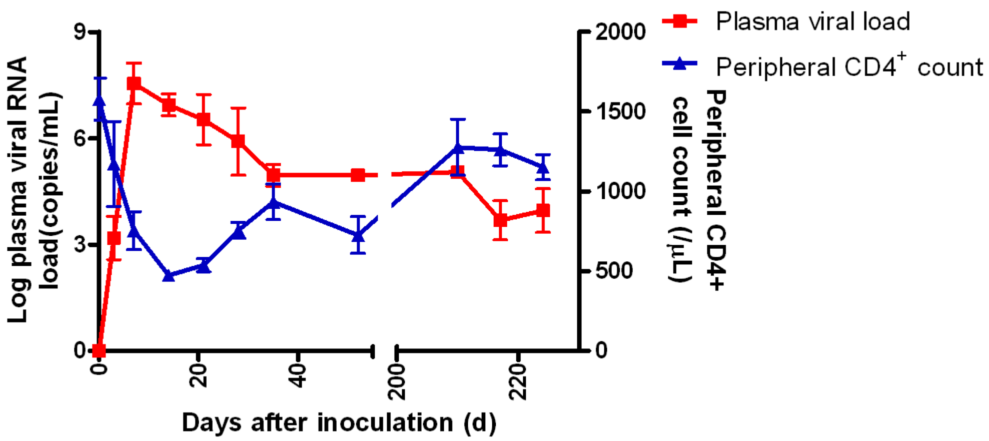

Plasma viral load
Peripheral CD4+ count
Log plasma viral RNA load(copies/mL)
Peripheral CD4+ cell count (/μL)
Days after inoculation (d)
9
6
3
0
2000
1500
1000
500
0
0
20
40
200
220
